# Design and Simulation of a Multi-Sheet Beam Terahertz Radiation Source Based on Carbon-Nanotube Cold Cathode

**DOI:** 10.3390/nano9121768

**Published:** 2019-12-12

**Authors:** Yifan Zu, Xuesong Yuan, Xiaotao Xu, Matthew T. Cole, Yu Zhang, Hailong Li, Yong Yin, Bin Wang, Yang Yan

**Affiliations:** 1 School of Electronic Science and Engineering, University of Electronic Science and Technology of China, Chengdu 610054, China; zuyf@std.uestc.edu.cn (Y.Z.); xxt@std.uestc.edu.cn (X.X.); lihailong@uestc.edu.cn (H.L.); yinyong@uestc.edu.cn (Y.Y.); wb@uestc.edu.cn (B.W.); yanyang@uestc.edu.cn (Y.Y.); 2 Department of Electronic and Electrical Engineering, University of Bath, North Road, Bath BA2 7AY, UK; m.t.cole@bath.ac.uk; 3 State Key Laboratory Optoelectronic Materials and Technologies, Sun Yat-sen University, Guangzhou 510275, China; stszhyu@mail.sysu.edu.cn

**Keywords:** nanotechnology, carbon nanotubes, cold cathode, field emission, terahertz, vacuum electronic devices, multi-sheet beam, high-order mode

## Abstract

Carbon nanotube (CNT) cold cathodes are proving to be compelling candidates for miniaturized terahertz (THz) vacuum electronic devices (VEDs) owning to their superior field-emission (FE) characteristics. Here, we report on the development of a multi-sheet beam CNT cold cathode electron optical system with concurrently high beam current and high current density. The microscopic FE characteristics of the CNT film emitter is captured through the development of an empirically derived macroscopic simulation model which is used to provide representative emission performance. Through parametrically optimized macroscale simulations, a five-sheet-beam triode electron gun has been designed, and has been shown to emit up to 95 mA at 3.2 kV. Through careful engineering of the electron gun geometric parameters, a low-voltage compact THz radiation source operating in high-order ${\mathrm{TM}}_{5,1}$ mode is investigated to improve output power and suppress mode competition. Particle in cell (PIC) simulations show the average output power is 33 W at 0.1 THz, and the beam–wave interaction efficiency is approximately 10%.

## 1. Introduction

Due to its wide bandwidth, good directionality, high spatial and temporal resolution, terahertz (THz) technology has attracted wide interest in a range of applications, such as high data rate communications, radar systems, electronic countermeasures, biomedical diagnostics, and security inspection [1,2,3,4,5,6]. Vacuum electronic devices (VEDs), one of the several existing technologies capable of generating THz waves, have received extensive attention due to their high output power and high electronic efficiency [7,8]. However, as operating frequencies continue to increase further toward the THz regime, significant and unavoidable physical limitations become functionally prohibitive for many generation approaches. The size of THz VEDs is usually small (<1 cm^3^), since the operating wavelength must be compatible with the dimensions of the high-frequency (HF) system, such as THz traveling wave tubes (TWTs) and THz extended interaction klystrons (EIKs) which normally have characteristic geometries of the order of a few mm [9,10].

As the device gets smaller, a smaller electron beam with high current is used to keep the beam diameter sufficiently smaller than the device operating wavelength. This is becoming increasingly difficult to engineer, particularly as almost all commercially available modern THz VEDs employ thermionic cathode electron guns. Due to their high operating temperature, thermionic cathodes must be kept at comparatively long working distances and be placed sufficiently distant from the HF system so as to not induce thermal damage. As a result, it has proven especially challenging for the electron beam to enter the HF system, which is necessary to achieve efficient beam–wave interaction required for THz generation. There is, as a result, a pressing need to produce small diameter, high current, precisely-aligned electron beams in order to solve the present limitations affecting THz VEDs performance [1]. The use of new materials, new manufacturing processes, integration procedures, and new emission mechanisms present one emerging approach to realizing such advanced electron emission topologies. Field-emission (FE) cold cathodes are an outstanding candidate for the replacement of incumbent thermionic electron sources in THz VEDs. FE sources readily overcome many of the inherent limitations associated with traditional thermionic devices, including their slow temporal response and high temperature operation [11,12,13,14]. Meanwhile, the gap between the emitter and the HF system will be dramatically shortened in a cold cathode electron optical system, thereby relaxing design difficulties associated with the need for precise alignment of the beam.

The collective contraction of the design also makes possible a smaller HF system. It would pose new challenges associated with increasing the output power. A solution for operating the circuit in high-order mode is adopted to overcome the frequency limitation and output power restriction caused by the small size of the HF system. In contrast to the fundamental mode commonly used, a high-order mode can support a larger-sized HF system for a given frequency [15]. However, multiple modes coexist in a large-sized HF system. In this report we reveal the significant challenges in achieving operational stability due to mode competition. Commonly there are multiple suitable beam–wave interaction locations within a higher-order mode HF system. If multiple electron beams are placed at multiple suitable beam–wave interaction locations, the mode purity of a higher-order mode circuit can be greatly improved. As a result, mode competition can be suppressed. Additionally, compared with traditional cylindrical electron beams, the broader transverse proportion in the sheet beam, developed here, provides a high current and increases the output power of the device substantially (>30%) [16].

Since the discovery of carbon nanotubes (CNTs), they have received wide attention as a promising FE cold cathodes material due to their high electrical conductivity, large aspect ratio, and excellent chemical and temporal stabilities [17]. They have unprecedented potential and have proven to be an enabling technology towards the manufacture of FE-based flat-panel displays, high-performance X-ray sources and, more recently, cold cathode VEDs [18,19,20,21,22]. As their emission current density increases significantly as the cathode size decreases, CNT based FE electron sources are very well suited for the required aggressive miniaturization needed for THz VEDs. Several attempts have been made to fabricate emitters using directly grown CNTs, CNT paste coatings on several substrates, super-aligned CNT yarns, and CNT films or CNT arrays transferred on sharp metallic tips [23,24,25,26,27,28]. Moreover, some progress has been made in experimental research using CNTs as cold cathode electron optical systems in THz vacuum tube sources [29,30]. However, there remains a significant challenge to scale the performance of many of these devices, especially when attempting to produce high current densities across large areas. Driven by end-user technology needs, recent research on the FE properties of CNT has focused primarily on lowering the threshold voltage, raising the emission current density and enhancing the long-term FE stability. It has been seen that enhancing the adhesive strength between the CNTs and their substrate is a direct method of dramatically improving the emission temporal stability of CNTs, particularly during challenging high current density emission. Compared to their inked or post-growth deposited counterparts, CNT electron emitters that are fabricated by advanced chemical vapor deposition (CVD) on catalytic substrates benefit from improved substrate adhesion and low contact resistance [31]. Yan et al. [32] fabricated and tested a CNT emitter formed as a CNT film grown directly on nickel chromium (Ni80Cr20) alloy wire using microwave plasma-enhanced chemical vapor deposition (PECVD), and obtained an extremely large emission current density of 7.65 A/${\mathrm{cm}}^{2}$ at a relatively low electric field of 2.13 kV/mm. Such a Ni80Cr20 alloy-supported CNT emitter is extremely well-suited for the proposed multi-sheet beam high-order mode THz radiation source in both emission geometry and physical dimension.

In this paper, a multi-sheet beam electron optical system based on the CNT film emitter is investigated, and a high-order mode compact THz radiation source is designed, which we hope will pave the way for the development of an emerging class of THz VEDs based on CNT cold cathode technologies. Using previous experimental results [32], we developed an FE empirical model based on CVD-synthesized, full-coverage CNTs. This was achieved through the extraction of equivalent parameters via numerical fitting across large sample sets in order to produce a corresponding macroscopic simulation model that accurately reflects the microscopic FE characteristics of standard CVD-synthesized, vertically aligned multi-walled CNT thin film emitters produced in our laboratory. Secondly, through parametrically optimized macroscale simulations, a five-sheet-beam triode electron gun based on the equivalent FE simulation model is proposed and is shown to enhance the emission performance significantly. Finally, combined with the electron gun, a high-order mode compact THz radiation source operating in the ${\mathrm{TM}}_{5,1}$ mode is designed in order to improve the output power whilst simultaneously suppressing mode competition.

## 2. Materials and Methods

To a large extent, the ability to generate high-emission current densities across large areas from CNT emitters is one of the key factors in solving the physical limitations of VEDs in modern THz applications. Toward this aim, it is vital to choose a suitable electron gun architecture that has the ability to simultaneously generate a high beam emission current and high-emission current density [33,34,35,36]. In designing the CNT cold cathode electron gun, it is important that such design tools take into consideration real, data-supported emission characteristics specific to the material from which the emission will be coming, CNTs in the present case [32]. Although it may be functionally possible to produce a nanometrically resolved CNT emitter model, the empirical validity of such approaches would still, nonetheless, require checking and the extremely small scale and high node number required may be overly time consuming and computationally infeasible in real computational times. Although direct emission site modelling pays an extremely significant role in producing computational tools that can approximate the emission of such multi-scalar systems, we must look towards new empirically driven approaches to capturing the emission characteristics of increasingly well established materials. Thus, in order to demonstrate the ability of CNT-based cold cathode guns for VEDs, the use of equivalent FE simulation models has been proven to be an extremely useful tool [21]. Given the CNTs’ quasi-metallic nature, the basic field emission process is suitably well described by a generalized Fowler–Nordheim (FN) equation of the form:

(1) $$J=A\times E^{2}\times exp\left(-B/E\right),$$

where *A* and *B* are the approximate FN constants, and which are estimated by numerical fitting based on previous experimental results. If the *A* and *B* constants are clearly obtained from a large population an equivalent, quasi-metallic CNT field emission system via experimentation, it is possible to establish a corresponding macroscopic simulation model with moderate accuracy that accurately reflects the microscopic, and technologically relevant, FE characteristics of the CNT film emitter.

**Figure 1:**
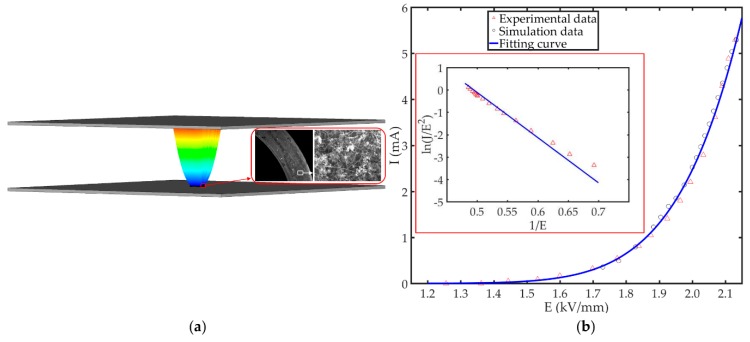
Typical emission characteristics of the carbon nanotube (CNT) emitters. (**a**) The field-emission (FE) properties of the CNT film emitter was determined using a simple diode configuration. Insets from Reference [32]: scanning electron microscope (SEM) images of CNTs grown on Ni80Cr20 alloy wire; (**b**) fitting, simulation and experimental emission current as a function of applied electric field. Inset: the corresponding Fowler–Nordheim (FN) plots presented in terms of *ln*(*J*/$E^{2}$) versus 1/*E*.

In the study of the CNT film emitters, prepared by Yan et al. [32], we found that the CVD-deposited CNTs greatly improved the emission current density compared to slurry based methods, which we attribute to the enhanced adhesion between the CNTs and the substrate. The direct synthesis of CNTs on self-catalyzing Ni80Cr20 wire substrates without any additional catalyst layers and the wrapping of the CNTs with a carbon nanoflake layer deposited on the surface of the wire substrate using microwave PECVD are responsible for enhanced adhesion. Scanning electron microscope (SEM) images of the Ni80Cr20 alloy wire covered with non-oriented CNT films are shown in the inset of Figure 1a [32]. Figure 1b shows the corresponding FE current-field performance of the emitter. Here, the CNT film emitter model is created using the particle in cell (PIC) solver and particle tracking solver in commercially available 3D simulation software (CST). Non-linear least-squares fitting is used to determine the FN *A* and *B* constants by engaging with a wide range of measurement samples. Applying our fitting procedure, we find $A=2.17\times 10^{-4}\;A/V^{2}$ and $B=2.02\times 10^{7}\;V/m$. As shown in Figure 1a, the FE characteristics of the emitter was studied in an ultrahigh-vacuum under a pressure of $1\times 10^{-7}$ Pa using a diode configuration. The distance between the CNT film emitter cathode and the anode was 2 mm. The active FE area was calculated to be about $6.9\times 10^{-4}\;{\mathrm{cm}}^{2}$. We find that our experimental and simulation data align very well, demonstrating a maximum operating emission current density of $7.65\;A/{\mathrm{cm}}^{2}$ at 2.13 kV/mm, which is the highest point in Figure 1b. Figure 1b shows typical experimental and simulated emission current (I) profiles as a function of the applied electric field (E). Using the extracted constants in our PIC simulations, our models suggest an equivalent electric field dependent emission with less than a 1% deviation between the model and the experimental data, for all electric fields considered in this study.

## 3. Application and Results

### 3.1. Sheet-Beam Electron Optical System Based on the Carbon Nanotube (CNT) Film Emitter

**Figure 2:**
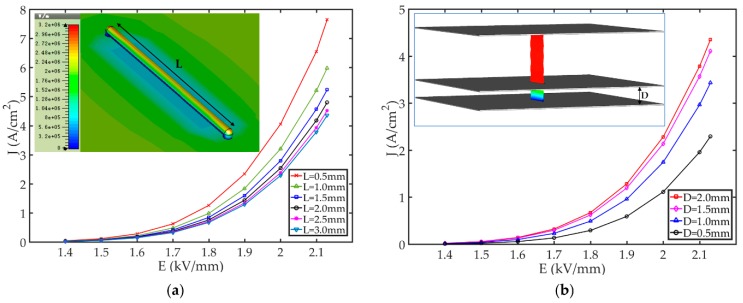
The grid triode system of a single CNT film emitter FE. (**a**) FE current density as a function of the effective length of the CNT film emitter. Inset: The surface electric field distribution of the emitter and cathode substrate when *L* = 3 mm; (**b**) FE current density as a function of the distance between the CNT film emitter and the anode. Inset: beam trajectories.

The high-emission current density ($7.65\;A/{\mathrm{cm}}^{2}$) at the relatively low threshold field (2.13 kV/mm), made the novel Ni80Cr20 wire alloy CNT film emitter a suitable selection for the present cathode. In order to further maximize the emission current necessary to achieve the functional requirements to initiate oscillation currents in VEDs, we must consider extending the length of Ni80Cr20 alloy wire so as to increase the cathode emission area. At the same time, due to the non-uniformity of the electric field on the surface of the nominally cylindrical wire cathode, it is essential that we undertake a multi-scalar analysis of the CNT emitter. A key function of the present simulator is to analyze and optimize the emitter structure. Based on the classical parallel-diode configuration used in the experiment, a gridded triode sheet beam electron optical system based on the CNT film emitter has been designed. The gridded triode system is composed of a cathode substrate, a CNT film emitter, a grid, and an anode, as illustrated in the inset of Figure 2b. The CNT film emitter is fixed via an electrical contact to the cathode substrate. The grid is coaxial with the anode and cathode substrate and controls the extraction of electrons from the CNT film emitter. Using the particle-tracking solver, the effect of electromagnetic fields on the movement of charged particles can be calculated. Based on electrostatic and magnetostatic fields resulting from static sources, particles are tracked through the computational domain, as shown in the inset of Figure 2b. Due to the influence of the grid and the CNT curved film, it is highly improbable that the cathode emitter surface emits to the same extent simultaneously across its entire surface. The effective length of the CNT film emitter is *L*. The diameter of the patterned CNT film emitter is about 0.1 mm. The distance between the CNT film emitter and the grid is *D*. In order to reduce the influence of the grid hole on the surface electric field of the cathode, and at the same time retain the grid’s high beam transparency, the dimension of the grid hole is set to 0.18 mm × 3.2 mm. Figure 2a shows the effect of *L* on the emission characteristics when *D* = 2 mm. It indicates that the emission current density decreases *L* increases, which is due to the non-uniformity of electric field on the surface of the CNT film emitter. As *L* increases, the CNT film emitter changes from the tip-dominated emission to large area sheet emission as additional emission sites become excited and as the electric field on the surface of the emitter becomes increasingly uniform due to burn-out and aging processes, excluding the highly non-uniform ends of the emitter. As *L* continues to increase the influence of electric field non-uniformities on the surface of the CNT film emitter decreases, and the emission current density remains substantially unchanged. The inset of Figure 2a shows the surface electric field distribution of the emitter and cathode substrate when *L* = 3 mm. Figure 2b shows the effect of *D* on the emission characteristics when *L* = 3 mm. When *D* is small, the effect of the grid on the surface electric field of the emitter is intensified, and the emission current at the center of the grid hole is reduced due to the lower effective electric field. However, the operating voltage can be reduced by shortening the distance between the grid and the emitter, which beneficially reduces the likelihood of vacuum gap breakdown.

**Figure 3:**
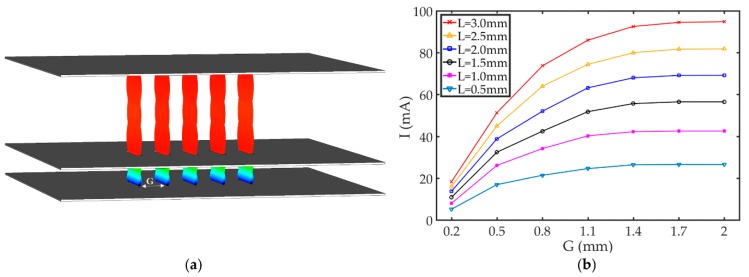
The five-sheet-beam electron optical system based on the CNT film emitter. (**a**) Simulated beam trajectories; (**b**) the FE current as a function of *L* and *G*.

To further study the effects of increasing the emission current, array arrangements of CNT film emitters were explored following on from reported dramatic improvements in emission characteristic by Li et al. [37] and Chouhan et al. [38]. A five-sheet-beam electron optical system based on the CNT film emitter was proposed, as shown in Figure 3. Five parallel CNT film emitters were fixed on the cathode substrate, as shown in Figure 3a. The distance between two parallel CNT film emitters was *G*. In order to reduce the probability of breakdown, and at the same time to obtain a larger emission current density, the distance between the grid and the CNT film emitters was minimized to 1.5 mm. According to previous experiments [32], we have found that significant vacuum breakdown occurs when the surface electric field of the CNT film emitter is larger than 2.15 kV/mm. The grid-anode voltage was, therefore, set to 3.2 kV to ensure high-emission currents without inducing breakdown. All other parameters remained the same as per the above single gridded triode system. Due to shielding effects between neighboring CNT film emitters, the *G* is an important macroscale parameter for the optimization of the array emission performance. Figure 3b shows the FE current as a function of *L* and *G*. As *G* increased, we find that the shielding effect between neighboring emitters was weakened and the emission current gradually increased. When *L* was longer, the emission current increased more obviously, suggesting that longer emitters experienced grater shielding. The emission current reached a maximum and remained stable at *G* ≥ 1.7 mm.

### 3.2. 0.1 THz Five-Sheet Beam High-Order Mode Interaction Circuit

**Figure 4:**
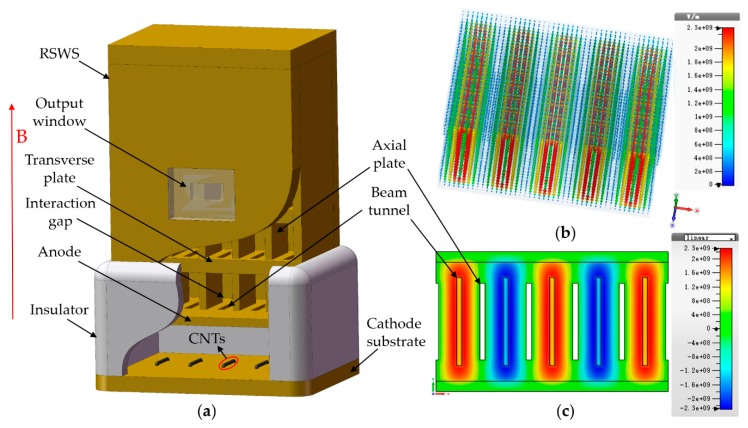
The five-sheet-beam terahertz (THz) radiation source based on CNT cold cathode. (**a**) Structure of the interaction circuit; (**b**) electric field arrows distribution of ${\mathrm{TM}}_{5,1}$ mode; (**c**) $E_{z}$-field contours at *z* = 0 plane of ${\mathrm{TM}}_{5,1}$ mode.

The extended interaction oscillator (EIO) is one of the most established VED sources for the emission of THz radiation. In the low frequency THz range (<0.7 THz), EIO has far reaching advantages over solid-state devices, photonic devices, and other traditional microwave tubes, chief amongst which is their extremely high output powers that can in excess of the watt level [39]. A high-order mode EIO was designed for the aforementioned five-sheet beam electron optical system based on the CNT film emitter. The 3-D model of the interaction circuit is shown in Figure 4. It was composed of a resonant slow wave structure (RSWS), an output window, a five-sheet-beam CNT cold cathode electron gun, and insulators. The present RSWS is a periodic standing-wave circuit in the longitudinal direction. The axial mode was chosen as 2π mode as it provided equal direction to the electric field at each gap for enhanced modulation of the electron beam [40]. Established beam-wave interaction theory shows that the electron beam interacts with the $E_{z}$ component of the electric field [15]. Therefore, the transverse mode is termed ${\mathrm{TM}}_{n,1}$, where the index *n* is determined by the variation in the distribution pattern of the axial electric field along the transverse direction. The RSWS designed in this study consisted of rectangular interaction gaps, sheet beam tunnels and coupling cavities in the different sides, as depicted in Figure 4a. The generated electromagnetic wave in the resonant cavity was extracted through a standard WR-8 waveguide output window attached to one of the coupled cavities with a coupling hole. The eigenmode solver in CST was used to simulate the cold cavity of the RSWS. The transverse mode was chosen as the ${\mathrm{TM}}_{5,1}$ mode, and the axial mode was the 2π mode. The resonant frequency was 100.56 GHz. The electric field distribution of the cold cavity is shown in Figure 4b. There are five parts of the $E_{z}$ field distributing along the x-direction with the ${\mathrm{TM}}_{5,1}$ mode, as shown in Figure 4c. Between adjacent parts are zero-field points. Five sheet beam tunnels pass through the rectangular interaction gaps and are located at the maximum $E_{z}$ field strength of each gap. Since the interaction circuit operates in a high-order mode, it experiences notable mode competition. By placing the metal plate at zero electric field of higher order modes and optimizing multi-beam tunnels, it is possible to establish a positive field with the ${\mathrm{TM}}_{5,1}$ mode for effective beam-wave interaction, and a weaker field with the fundamental mode or possible competition modes due to the electric wall characteristics of the conductors, thereby suppressing the generation of the fundamental mode and reducing the deleterious effects of mode competition. The operating parameters of the interaction circuit are listed in Table 1. According to the actual structural parameters listed in Table 1, the cavity size of the EIO was calculated to be about 8.7 mm × 4.0 mm × 5.5 mm.

**Figure 5:**
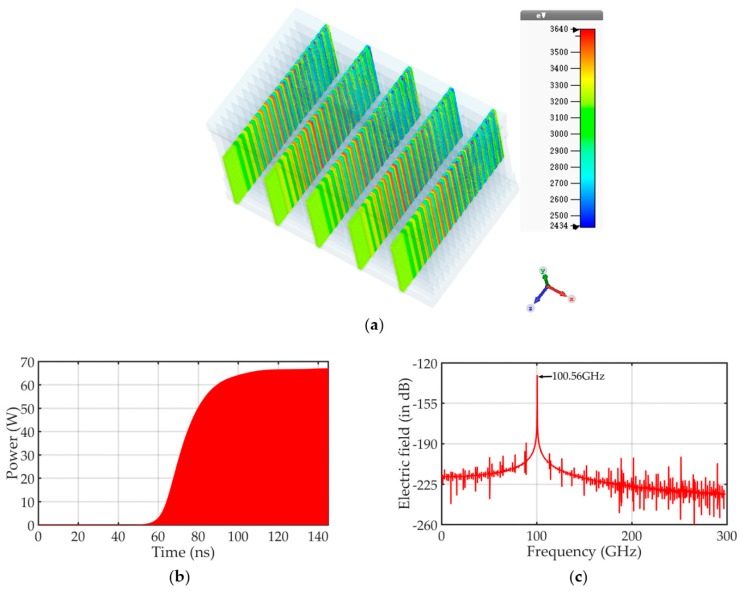
Particle in cell (PIC) results depicting gun operation following beam–wave interaction stability with operation voltage 3.2 kV, showing (**a**) notable electron bunch occurring; (**b**) signal power amplitude at the output port; (**c**) output signal spectrum showing high mode purity at 100.56 GHz.

The performance of the designed EIO circuit has been analyzed using the CST PIC solver. The PIC solver calculates the development of fields and particles through time at discrete time samples [41]. According to the five-sheet beam electron optical system proposed above, five uniform sheet beams excited at 3.2 kV, producing a net current of 95 mA, are injected into the device, as shown in Figure 5a. A constant magnetic field of 0.60 T is applied for beam focusing. As shown in Figure 5b,c, an average output power of 33 W is obtained at 100.56 GHz, and the beam–wave interaction efficiency is approximately 10%. With the beam–wave interaction between the electron beam and the specified frequency of the electromagnetic wave, electron bunching occurs in the RSWS, as shown in Figure 5a. Figure 5a shows the device stably operates in the ${\mathrm{TM}}_{5,1}$ mode without mode competition. Here, a highly compact, high-order mode THz EIO can be effectively realized using the CNT film emitter as a multi-sheet beam emission system.

## 4. Conclusions and Discussion

In the development of vacuum FE electronics in the regions of short millimeter waves and the THz regime, CNTs are one of the very few promising materials capable of producing the required high-emission current during cold cathode emission. In this paper, a multi-sheet beam electron optical system based on arrays of CNT film emitters is studied towards the development of an emerging class of novel THz VEDs. The microscopic FE characteristics of the CNT film emitter is reflected by establishing a macroscopic simulation model. Through parametrically optimized macroscale simulations, a five-sheet beam triode electron gun has been realized. Simulation results show that a total of 3.2 kV/95 mA of five-sheet electron beam is achieved at global electric fields as low as 2.13 kV/mm. In addition, a high-order mode-specific design for making a stably operated circuit of with ${\mathrm{TM}}_{5,1}$ mode is proposed in combination with the five-sheet beam electron gun design parameters. The ${\mathrm{TM}}_{5,1}$ mode has obvious advantages compared to other modes. Compared with lower order modes such as ${\mathrm{TM}}_{4,1}$ and ${\mathrm{TM}}_{3,1}$ mode, it has larger cavity power capacity and higher output power. Compared with higher order modes such as ${\mathrm{TM}}_{6,1}$ and ${\mathrm{TM}}_{7,1}$ mode, it has fewer competition modes, and it is easier to overcome mode competition. An average output power over 33 W is obtained at 100.56 GHz, and the device indeed works at ${\mathrm{TM}}_{5,1}$ mode without mode competition. The beam-wave interaction efficiency is about 10%. The low operation voltage, compact THz radiation source has the high power densities to date (370 W/cm^3^).

We provide a method for the design of CNT cold cathode electron guns, and further propose a solution for increasing the output power of VEDs, to develop an emerging class of CNT-based THz radiation sources. On the one hand, based on this method, a variety of electron optical systems using the CNT film emitters will be developed according to different requirements. In the present study, we have realized a multi-sheet-beam electron gun structure by extending the length of the Ni80Cr20 alloy wire and the linear array arrangement to enhance the emission properties. In principle, it is practical to improve the emission current of the CNT film emitter by these two effective methods to meet the requirements of the VEDs for the start-oscillation current. Further detailed consideration of changing the diameter of the Ni80Cr20 alloy wire to improve the emission performance, transforming the arrangement of the CNT film emitter to produce an annular sheet electron beam, and integrating an insulator layer between the emitter and the cathode substrate to enhance the field enhancement factor, will likely manifest in functional enhancements and improved manufacturability. On the other hand, we aim to further improve the operating frequency and output power as well as enhance the operational stability of the device, such as higher operating mode, more electron beams, more stringent parameter optimization and detailed theoretical analysis. In addition, we will consider integrating the electron gun inside the HF cavity to produce a pre-modulation electron beam to improve the beam–wave interaction efficiency and level of integration of the device, since electron emission can be modulated in principle by a HF field to effectively pre-bunch the electron beam for coherent electromagnetic wave generation.

## Figures and Tables

**Table 1:** Structural parameters.

Parameters	Values and Units
Interaction gap width	1.74 mm
Interaction gap height	4.00 mm
Interaction gap length	0.15 mm
Period length	0.32 mm
Electron beam tunnel width	0.18 mm
Electron beam tunnel height	3.20 mm
Distance between adjacent electron beam tunnels	1.74 mm
Operating frequency	100.56 GHz
Operating voltage	3.2 kV
Operating current	95 mA
Axial focusing magnetic field	0.60 T
